# Acceptability of self- collection for human papillomavirus detection in the Eastern Cape, South Africa

**DOI:** 10.1371/journal.pone.0241781

**Published:** 2020-11-10

**Authors:** Ongeziwe Taku, Tracy L. Meiring, Inger Gustavsson, Keletso Phohlo, Mirta Garcia-Jardon, Zizipho Z. A. Mbulawa, Charles B. Businge, Ulf Gyllensten, Anna-Lise Williamson

**Affiliations:** 1 Division of Medical Virology, Department of Pathology, Faculty of Health Sciences, University of Cape Town, Cape Town, South Africa; 2 Institute of Infectious Disease and Molecular Medicine, University of Cape Town, Cape Town, South Africa; 3 Department of Immunology, Genetics, and Pathology, Biomedical Center, Science for Life Laboratory Uppsala, Uppsala University, Uppsala, Sweden; 4 Department of Pathology at Walter Sisulu University and National Health Laboratory Service, Mthatha, South Africa; 5 SAMRC Gynaecological Cancer Research Centre, University of Cape Town, Cape Town, South Africa; 6 Department of Laboratory Medicine and Pathology, National Health Laboratory Service Nelson Mandela Academic Hospital and Walter Sisulu University, Mthatha, Eastern Cape, South Africa; 7 Medical Virology, National Health Laboratory Service, Nelson Mandela Academic Hospital, Mthatha, South Africa; 8 Department of Obstetrics and Gynaecology, Nelson Mandela Academic Hospital, Mthatha, South Africa; 9 Department of Obstetrics and Gynaecology, Faculty of Health Sciences, Walter Sisulu University, Mthatha, South Africa; Universidade Estadual de Maringa, BRAZIL

## Abstract

Human papillomavirus (HPV) testing on vaginal self-collected and cervical clinician-collected specimens shows comparable performance. Self-sampling on FTA cards is suitable for women residing in rural settings or not attending regular screening and increases participation rate in the cervical cancer screening programme. We aimed to investigate and compare high-risk (HR)-HPV prevalence in clinician-collected and self-collected genital specimens as well as two different HPV tests on the clinician collected samples. A total of 737 women were recruited from two sites, a community health clinic (n = 413) and a referral clinic (n = 324) in the Eastern Cape Province. Cervical clinician-collected (FTA cards and Digene transport medium) and vaginal self-collected specimens were tested for HR-HPV using the *hpVIR* assay (FTA cards) and Hybrid Capture-2 (Digene transport medium). There was no significant difference in HR-HPV positivity between clinician-collected and self-collected specimens among women from the community-based clinic (26.4% vs 27.9%, p = 0.601) or the referral clinic (83.6% vs 79.9%, *p* = 0.222). HPV16, HPV35, and HPV33/52/58 group were the most frequently detected genotypes at both study sites. Self-sampling for HPV testing received a high positive response of acceptance (77.2% in the community-based clinic and 83.0% in referral clinic). The overall agreement between *hpVIR* assay and HC-2 was 87.7% (k = 0.754). The study found good agreement between clinician-collected and self-collected genital specimens. Self-collection can have a positive impact on a cervical screening program in South Africa by increasing coverage of women in rural areas, in particular those unable to visit the clinics and women attending clinics where cytology-based programs are not functioning effectively.

## Introduction

Cervical cancer (CC), is the third-ranking cause of cancer disease with an estimated 569,847 cases leading to 311,365 deaths in women globally in 2018 [1]. The age-standardized incidence rate of cervical cancer varies worldwide from ≤2 to 75 per 100,000 women in different populations [2]. In South Africa, CC is the leading cause of cancer in women of reproductive age (15–44 years) and the most important cancer in black women. About 12,983 new cervical cancer cases are diagnosed annually in South Africa (estimates for 2018) with an incidence of 31.7 per 100, 000 women [3, 4].

CC is causally associated with infection by high-risk types of human papillomavirus (HPV) [1]. Cervarix, which targets HPV16 and 18 [5], is the current HPV vaccine used in school-based HPV vaccination programme in South Africa. Although this vaccine is expected to prevent at least 70% of CCs, both vaccinated women and unvaccinated women still need to continue to be screened for CC [5]. CC screening methods include Visual Inspection with Acetic acid (VIA), cytology-based (Pap smear or liquid-based cytology) and HPV DNA testing [6]. Worldwide, cytology-based screening is still the most used screening test, although it has not been successfully implemented in African countries, including South Africa [7, 8]. In the Eastern Cape province of South Africa CC screening coverage is <50%, which is below the national target of 70% coverage [9]. Pap smear screening is not readily available to most of the women in rural South Africa, including Eastern Cape Province [9, 10]. In addition, while CC screening programmes may be available in some local clinics, the majority of women are not aware of the available services and not likely to participate [11–13].

HPV DNA testing is an effective CC prevention screening method and offers many advantages compared to cytology-based screening. This includes high sensitivity in predicting future precancerous lesions and CC, ability to perform self-collection of specimen for HPV testing, and the possibility to increase of screening intervals for women with a HPV negative test (>5-years) [14, 15]. A randomized study in India reported that a single HPV DNA test is more likely to lower the mortality rate of CC disease as compared to cytology-based screening or VIA [16]. Australia and Netherlands have introduced vaginal self-sampling for HPV DNA testing in their routine screening program as an alternative screening method for non-attendees [17, 18]. and shown increased participation [19–21]. Therefore, introducing a self-collection method might overcome barriers that limit the use of the Pap smear test and make CC screening accessible to more women.

Liquid-based transport medium is commonly used to transport and store cervical or vaginal specimens for HPV testing. FTA cards for storage of cervical or vaginal specimens, have several advantages over a liquid-based media. These include inclusion of a color indicator to confirm that the sample is applied correctly, the samples become non-infectious once dried, long term stable storage at room temperature and easy transport [22–25]. Unlike the liquid-based transport medium, FTA cards are suitable for self-collection at home and can be transported via regular mail or other means not involving a cold-chain [22]. The process of extracting DNA from the FTA card is easy, quick and can be automated [22, 24]. Using the FTA card in combination with a clinically validated HPV DNA test has also been shown to work effectively for detection of HR-HPV infection [26]; with clinical specimens depicting up to 100% agreement of HR-HPV detection between FTA cards and liquid-based medium [27].

Due to the lack of infrastructure to perform Pap smear tests, cytology screening is not fully functional in rural settings. It is essential to implement a CC screening test that is easily accessible (such as self-collection) and simple to handle for women that do not have access to healthcare facilities offering regular cytology screening. In this study we aim to a) determine the prevalence of HR-HPV genotypes in women attending a community-based clinic and a referral clinic in the Eastern Cape, b) investigate and compare the HR-HPV prevalence in clinician-collected and self-collected genital specimens on FTA cards and to c) investigate attitudes to self-collection in women from the Eastern Cape.

## Materials and methods

### Study population and sample collection

This cross-section study was carried out from September 2017 to March 2019 at a community health clinic and the referral clinic for women with abnormal Pap smears within the OR Tambo district in the Eastern Cape Province of South Africa. A total of 413 women age ≥30 years attending the community health clinic for CC screening or others reasons were recruited. Also, 324 women age ≥18 years with abnormal cervical cytology and CC were recruited from the referral clinic. Written signed consent forms were obtained from all study participants, and sociodemographic data with other factors were obtained through a questionnaire. The ethical approval for this study was granted by the Human Research Ethics Committees of the University of Cape Town (UCT) (HREC reference 615/2017), Walter Sisulu University (reference 090/2016), and Eastern Cape Department of Health Ethics (EC reference 2017RP0_484).

Study participants provided three genital specimens. One clinician-collected cervical and one self-collected vaginal specimen were collected using Viba-brushes (Rovers Viba-Brush, Rovers Medical Devices) and applied onto indicating FTA elute cards (GE Health Care Life Sciences, Buckinghamshire, United Kingdom). A second clinician-collected cervical specimen was obtained using cervical brushes and stored in Digene transport medium (Qiagen, Gaithersburg, MD, USA). All specimens were kept at room temperature until shipped to the UCT HPV laboratory for analysis. Among participants recruited from the referral clinic, a cervical biopsy was collected and sent to the National Health Laboratory Service for histological analysis. The histology results were interpreted according to the guidelines of the International Agency for Research on Cancer [28].

### Elution of DNA from FTA cards

DNA elution was performed as previously described [22]. Briefly, four 3.2 mm punches were obtained from each FTA card using the DBS Puncher Instrument (Perkin Elmer Life and Analytical Sciences, Wallac, Oy, Finland) and collected in a 96-well plate. The punches were washed with sterile water and the DNA eluted in 70 μl water by incubating in a PCR machine at 95°C for 30 minutes. The DNA was stored at -20°C until further use.

### Identification of HR-HPV using *hpVIR* real-time PCR

The eluted DNA from specimens on FTA cards was tested for HR-HPV using the clinically validated *hpVIR* real-time PCR assay [29]. *hpVIR* assay detects 12 HR-HPV genotypes (HPV16, 18, 31, 33, 35, 39, 45, 51, 52, 56, 58 and 59) and the analysis was performed as previously described [30]. Some HR-HPV types (16, -31, -35, -39, -51, -56, and -59) are identified as individual types, while HPV18 and -45, and HPV33, -52 and -58 are detected as two groups. The *hpVIR* assay also detect a human single copy house-keeping gene encoding Homo sapiens hydroxymethylbilane synthase (HMBS; GenBank accession no.M9523.1). This serves as a control for the integrity of the eluted DNA and interpretation of the results for HPV-negative samples. The *hpVIR* assay has a cut-off of for HMBS single copy gene, and 10 copies per PCR of HPV for a positive HR-HPV type [29].

### Detection of HR-HPV using Hybrid Capture-2

Cervical specimens in Digene transport medium were tested for 13 HR-HPV types (HPV16, 18, 31, 33, 35, 39, 45, 51, 52, 56, 58, 59 and 68) using the Hybrid Capture-2 (HC-2) assay (Qiagen Inc., Gaithersburg, MD; USA) according to the manufacturers protocol. A ratio of relative light units/cut-off ≥1 was considered positive while a ratio <1 was considered negative for HR-HPV types.

### Statistical analysis

Data analysis was performed using GraphPad Prism version 6 for Windows (Graphpad Software, La Jolla California USA). Descriptive statistics (medians and interquartile range) and frequency distribution were used to describe the sociodemographic and other variables of study participants in the population. For statistical analysis, chi-squared test was used to determine the difference in estimated HR-HPV prevalence between self-collected and clinician-collected samples. A p-value <0.05 was considered significant. The overall agreement and HR-HPV genotype distribution agreement (percent agreement, kappa values with 95% Confidence Intervals) were determined using the kappa statistic. The kappa values were interpreted using a standard method (http://www.graphpad.com/quickcalcs/Kappa2.cfm).

## Results

### Description of study participants

Table 1 summarises the baseline demographics of study participants and other factors. Women from the community-based clinic had a median age of 46 years (IQR: 38–55), most were ≥50 years (42.4%), 37.3% were HIV-positive and the majority had been pregnant (96.6%). Almost half the women had ≥3-lifetime sexual partners (49.4%) and most of the women never smoked (94.4%). A high proportion of women had attended high school or university (73.6%), and the monthly household income was <$139,36 (71.2%).

**Table 1:** Demographic and behaviour characteristics of study participants.

	Community clinic	Referral clinic
Variables	% (n/N)	% (n/N)
**HIV Status**		
No	62.7 (259/413)	27.8 (90/324)
Yes	37.3 (154/413)	71.3 (231/324)
Missing	0.0 (0/413)	0.9 (3/324)
**If yes, ARV’s?**		
No	3.9 (6/154)	1.3 (3/231)
Yes	96.1 (148/154)	98.3 (227/231)
Missing	0.0 (0/154)	0.4 (1/231)
**Age categories**		
18–29 years	……	13.0 (42/324)
30–39 years	31.5 (130/413)	33.3 (108/324)
40–49 years	26.2 (108/413)	31.2 (101/324)
≥50 years	42.4 (175/413)	22.5 (73/324)
Missing	0.0 (0/413)	0.0 (0/324)
**Highest level of education attained**		
Never/ primary	26.4 (109/413)	45.7 (148/324)
High school/university	73.6 (304/413)	54.3 (176/324)
Missing	0.0 (0/413)	0.0 (0/324)
**Household income**		
< $139,36	71.2 (294/413)	77.5 (251/324)
≥ $139,36	27.6 (114/413)	21.3 (69/324)
Missing	1.2 (5/413)	1.2 (4/324)
**Smoking status**		
Never	94.4 (390/413)	92.0 (298/324)
Former/current smoker	5.6 (23/413)	7.7 (25/324)
Missing	0.0 (0/413)	0.3 (1/324)
**Ever drank alcohol**		
No	91.0 (376/413)	81.5 (264/324)
Yes	9.0 (37/413)	18.2 (59/324)
Missing	0.0 (0/413)	0.3 (1/324)
**Age at first sexual experience**		
<16 years	12.1 (50/413)	19.4 (63/324)
16–18 years	52.5 (217/413)	54.3 (176/324)
≥18 years	34.9 (144/413)	26.2 (85/324)
Missing	0.5 (2/413)	0.0 (0/324)
**Lifetime sexual partners**		
1	20.3 (84/413)	15.1 (49/324)
2	30.0 (124/413)	23.8 (77/324)
≥3	49.4 (204/413)	60.8 (197/324)
Missing	0.2 (1/413)	0.3 (1/324)
**Used condoms during last sexual intercourse**		
No	70.0 (289/413)	65.4 (212/324)
Yes	29.1 (120/413)	33.6 (109/324)
Missing	0.9 (4/413)	1.0 (3/324)
**Method of contraceptive, last time had sex**		
None/implant/ligation etc	51.1 (211/413)	52.2 (169/324)
Injectables/birth control pill	21.1 (87/413)	20.7 (67/324)
Condoms	26.1 (108/413)	26.9 (87/324)
Missing	1.7 (7/413)	0.3 (1/324)
**Using any contraception currently**		
No	61.7 (255/413)	60.8 (197/324)
Yes	37.1 (153/413)	38.6 (125/324)
Missing	1.2 (5/413)	0.6 (2/324)
**Pregnancy**		
No	3.2 (13/413)	4.6 (15/324)
Yes	96.6 (399/413)	95.1 (308/324)
Missing	0.2 (1/413)	0.3 (1/324)

**HIV**: human immunodeficiency virus **ARV’**s: Antiretrovirals, **n**: number of women responsed, **N**: total number of study participants; **Missing**: not answered

Median age of the referral clinic women was 40.5 years (IQR: 33–49) and the majority were HIV-positive (71.3%). Half of the study participants had attended high school or university (54.3%) and 77.4% of women had a monthly household income of <$139,36. The majority of women had their first sexual experience at the age of 16–18 years (54.3%), with 60.8% of them having ≥3-lifetime sexual partners.

### HR-HPV prevalence and agreement between self-collected and clinician-collected specimen using *the hpVIR assay*

All specimens on FTA cards had sufficient amounts of genomic DNA (10 copies or more of the HMBS single copy gene) for the HPV test to be informative. For women recruited at the community-based clinic, there was no significant difference in HR-HPV prevalence between self-collected and clinician-collected samples [27.9%(115/413) vs 26.4%(109/413), *p* = 0.639]. HR-HPV positivity between self-collected and clinician-collected samples showed an agreement of 86.9%, with a kappa value of 0.669 [95%CI: 0.588–0.750, (Table 2)].

**Table 2:** Concordance between high-risk HPV in self-collected and clinician-collected genital specimens from the community and the referral clinics.

**Community clinic**	**Self-collected**				
		**HR-HPV positive (%)**	**HR-HPV negative (%)**	**Total (%)**	**% Agreement**
**Clinician-collected**	**HR-HPV positive (%)**	85 (20.6)	24 (5.8)	109 (26.4)	86.9 (k = 0.669)
	**HR-HPV negative (%)**	30 (7.3)	274 (66.3)	304 (73.6)	
	**Total (%)**	115 (27.9)	298 (72.1)	413 (100.0)	
**Referral clinic**	**Self-collected**				
		**HR-HPV positive (%)**	**HR-HPV negative (%)**	**Total (%)**	**% Agreement**
**Clinician-collected**	**HR-HPV positive (%)**	251 (77.4)	20 (6.2)	271 (83.6)	91.4 (k = 0.711)
	**HR-HPV negative (%)**	8 (2.5)	45 (13.9)	53 (16.4)	
	**Total (%)**	259 (79.9)	65 (20.1)	324 (100.0)	

**HR-HPV**: high-risk human papillomavirus, **k**-kappa value

Among the referral clinic women, HR-HPV prevalence was slightly higher in clinician-collected versus self-collected specimens, but the difference was not significant [83.6% (271/324) vs 79.9% (259/324), *p* = 0.222]. The observed overall agreement of HR-HPV infection between self-collected and clinician-collected samples was 91.4% [k = 0.711; 95%CI: 0.610 to 0.811, (Table 2)].

### HR-HPV genotypes and agreement between clinician-collection and self-collection using the *hpVIR assay*

**Fig 1:**
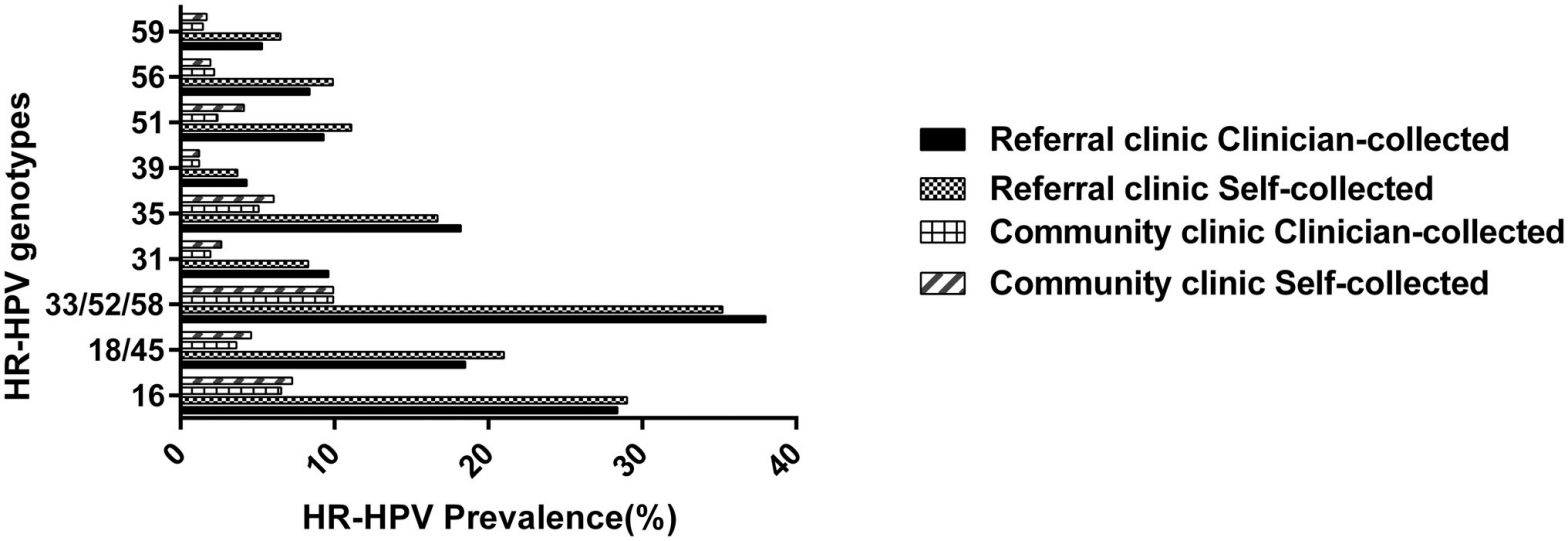
Prevalence of HR-HPV genotypes in self-collected and clinician-collected specimens using the hpVIR assay in the community and the referral clinics.

Of the 413 women attending the community-based clinic, 19.1% of the self-collected and 20.4% samples of clinician-collected samples were infected with single HR-HPV types, and this difference was not statistically significant (*p* = 0.601, Table 3). The prevalence of multiple HR-HPV infections was not significantly different between self-collected and clinician-collected specimens (8.7% vs 5.8%, *p* = 0.108). The most frequently detected HR-HPV genotypes were HPV16, HPV35, and the HPV33/52/58 group, both in self-collected and clinician-collected specimens (Fig 1). The agreement in the HR-HPV genotypes identified in self-collected and clinician-collected specimens ranged from moderate to almost perfect (k = 0.571–0.888), with HPV39 (99.8%, k = 0.888) and HPV31 (99.3%, k = 0.838) showing almost perfect agreement (Table 4).

**Table 3:** Comparison of self-collected and clinician-collected samples from women with single HR-HPV infections and multiple HR-HPV infections from the community and the referral clinics.

	Community clinic			Referral clinic		
Variables	Clinician-collected	Self-collected	p-value	Clinician-collected	Self-collected	p-value
Single infection	20.4% (85/413)	19.1% (79/413)	0.601	46.0% (149/324)	42.3% (137/324)	0.342
Multiple infection	5.8% (24/413)	8.7% (36/413)	0.108	37.7% (122/324)	37.7% (122/324)	1.000

**Table 4:** Concordance for HR-HPV genotypes between self-collected and clinician-collected specimen in the community clinic and the referral clinics.

	Community clinic			Referral clinic		
HR-HPV types	% Agreement	kappa value	95% CI	% Agreement	Kappa value	95% CI
16	96.4	0.717	0.581–0.853	89.2	0.735	0.653–0.817
18/45	96.6	0.571	0.369–0.773	90.7	0.705	0.606–0.803
33/52/58	94.0	0.658	0.533–0.782	86.1	0.701	0.670–0.782
31	99.3	0.838	0.659–1.000	96.3	0.780	0.659–0.900
35	96.6	0.678	0.519–0.836	95.4	0.841	0.763–0.919
39	99.8	0.888	0.669–1.000	96.9	0.627	0.413–0.840
51	97.8	0.629	0.406–0.853	93.5	0.660	0.525–0795
56	98.8	0.700	0.449–0.950	96.6	0.801	0.687–0.915
59	99.0	0.709	0.438–0.981	97.5	0.777	0.627–0.926

**HR-HPV**: high-risk human papillomavirus, **CI**: confidence intervals

In the referral clinic, the distribution was similar when comparing HR-HPV genotypes in self-collected and clinician-collected samples. The HPV33/52/58 group, HPV16 and HPV35 were the most frequently detected HR-HPV genotypes in both collection groups (Fig 1). The prevalence of single HR-HPV infection was somewhat higher in clinician-collected specimens as compared to self-collected specimens, but the difference was not statistically significant [46.0% vs 42.3%, *p* = 0.342, (Table 3)]. The HR-HPV prevalence of multiple infection was the same in self-collected and clinician-collected specimens [37.7% vs 37.7%, *p* = 1.000, (Table 3)]. The concordance of the HR-HPV genotype distribution between collection groups showed kappa values ranging between 0.627–0.841 (Table 4). The HPV16 genotype showed a substantial agreement between self-collected and clinician-collected samples (89.2%, k = 0.735, Table 4), while HPV35 showed an almost perfect agreement between self-collected and clinician-collected specimens (95.4%, k = 0.841).

### HR-HPV genotypes according to histology using *hpVIR* assay

Of the 324 women, histology results were available for 291 women including 2.1% (6/291) with inconclusive results (Table 5). More than half of the women had CIN3 (51.2%, 149/291), followed by CIN2 (23.0%, 67/291), no atypia (15.5%, 45/291), CIN1 (4.1%, 12/291) and CC (4.1%, 12/291). Overall, there was no significant difference in HR-HPV prevalence based on histology between self-collected and clinician-collected samples [78.4% (228/291) vs 81.8% (238/291), p = 0.299]. HR-HPV vaccine types (HPV16/18/31/33/45/52/58) were identified in 64.4% vs 46.7% of no atypia, 41.7% vs 41.7% of CIN1, 65.7% vs 59.7% of CIN2, 71.8% vs 71.1% of CIN3 and 75.0% vs 66.7% of CC between clinician-collected and self-collected, respectively (Table 5). The detection rate of the most commonly detected HPV genotypes (HPV16 and -35) was similar between self-collected and clinician-collected samples from women with CIN2/3 and CC [29.0% (66/228) vs 28.5% (65/228) and 19.7% (45/228) vs 19,3% (44/228), respectively]. Three women with CIN3 tested positive for HPV16 in their self-collected samples and were infected with multiple HPV types, but were negative in their clinician-collected samples. The HR-HPV copy number for three self-collected CIN3 samples were above the cut-off for HR-HPV positivity, but two of the samples had a low viral load.

**Table 5:** Distribution of HR-HPV types between self-collected and clinician-collected specimen according to histology results in women from referral clinic.

Histology results										
	NILM (n = 45)		CIN1 (n = 12)		CIN2 (n = 67)		CIN3 (n = 149)		Cervical cancer (n = 12)	
HR-HPV types	Clinician-collected	Self-collected	Clinician-collected	Self-collected	Clinician-collected	Self-collected	Clinician-collected	Self-collected	Clinician-collected	Self-collected
16	11	10	0	2	18	17	41	44	6	5
18/45	7	8	1	1	16	19	23	29	3	3
33/52/58	12	9	2	3	19	19	66	62	3	2
31	5	3	2	2	4	6	18	15	0	0
35	6	6	2	1	14	15	29	28	1	2
39	2	0	1	0	2	2	5	8	0	0
51	1	4	1	1	4	7	17	16	1	1
56	4	3	1	1	5	7	12	14	2	2
59	4	3	0	0	4	4	6	13	1	1
**HPV multiple infection**	14	12	3	4	19	22	61	65	5	4
**HR-HPV16/18/31/33/45/52/58**	29	21	5	5	44	40	107	106	9	8
**HR-HPV negative**	13	17	5	5	14	15	20	25	1	1
**HR-HPV positive**	32	28	7	7	53	52	129	124	11	11

**NILM**: negative for intraepithelial lesions or malignancy, **CIN**: cervical intraepithelial neoplasia, **HR-HPV**: high-risk human papillomavirus

### Acceptance of self-collection of specimens for HPV testing

The participants from both study sites gave a similar response regarding self-collection versus clinician-collection of specimen (Table 6). More than half of the women find self-collection and clinican-collection to be interesting (64.4% vs 66.1% in the community-based clinic and 54.0% vs 59.3% in referral clinic, respectively). A very small proportion of women from the community-based clinic experienced discomfort when collecting the sample, both using self-collection (3.4%) and clinician-collection (3.9%). In the referral clinic, a slightly higher proportion of women experienced discomfort with clinician-collection (9.5%) as compared to self-collection (4.3%) of samples. At both study sites older women reported to be embarrassed and were more likely to have had only one Pap smear screening test in their lifetime [73.3% (22/30) self-collected vs 66.7% (20/30) clinician-collected at community-based clinic and 66.1% (39/59) self-collected vs 67.9% (36/53) clinician-collected at referral clinic]. Most participants at both study sites reported that they would be willing to perform self-collection at home and return the card for testing [77.2% in the community-based clinic and 83.0% in referral clinic, (Table 7)]. Given a choice, almost all participants preferred clinician-collection to self-collection of samples (95.6% in community-based clinic and 90.7% in referral clinic, Table 7).

**Table 6:** Experience of the mean at the with self-collection and clinician-collection.

	Community clinic		Referral clinic	
Variable	Clinician-collected %(n/N)	Self-collected %(n/N)	Clinician-collected %(n/N)	Self-collected %(n/N)
Embarrassed	12.8% (53/413)	14.0% (58/413)	16.4% (53/324)	18.2% (59/324)
Self-confident	17.0% (70/413)	16.0% (66/413)	16.7% (54/324)	16.7% (54/324)
Discomfort	3.9% (16/413)	3.4% (14/413)	9.5% (31/324)	4.3% (14/324)
Interested	64.4% (266/413)	66.1% (273/413)	54.0% (175/324)	59.3% (192/324)

**Table 7:** Investigation of the acceptability of self-collection for HPV testing.

	Community clinic	Referral clinic
Variables	% (n/N)	% (n/N)
**Would you be willing to collect a sample yourself (self-sample) at home and bring it in for testing?**		
Yes	77.2% (319/413)	83.0% (269/324)
Not sure	18.6% (77/413)	10.8% (35/324)
No	1.9% (8/413)	4.3% (14/324)
**If no, why not?**		
I prefer the specimen to be taken by the nurse	12.5% (1/8)	12.5% (1/14)
I will not be able to take the specimen correctly	62.5% (5/8)	62.5% (5/14)
**Which method do you prefer for cervical cancer screening**		
To take a sample myself	1.4% (6/413)	3.7% (12/324)
For a healthcare worker to take a sample	95.6% (395/413)	90.7% (294/324)
Either for myself to take a sample or a health worker to take a sample	1.2% (5/413)	2.5% (8/324)

### HPV detection in cervical clinician-collected specimen using the *hpVIR* assay and HC-2

A total of 628 women were screened for HR-HPV infection using HC-2 and the *hpVIR* assay. Overall, the HR-HPV prevalence was 46.2% using *hpVIR* compared to 48.3% using HC-2, with an agreement of 87.7% (k = 0.754, 95%CI;0.703–0.806) between the two assays (Table 8).

**Table 8:** Concordance of HR-HPV infection between the *hpVIR* and the HC-2 assays.

	*hpVIR*				
		HR-HPV positive (%)	HR-HPV negative (%)	Total (%)	% Agreement
**HC-2**	**HR-HPV positive (%)**	258 (41.1)	45 (7.1)	303 (48.3)	87.7 (k = 0.754)
	**HR-HPV negative (%)**	32 (5.1)	293 (46.7)	325 (51.7)	
	**Total (%)**	290 (46.2)	338 (53.8)	628 (100.0)	

**HR-HPV**: high-risk human papillomavirus, **k**-kappa value

## Discussion

The present study assessed the prevalence of HR-HPV genotypes between self-collected and clinician-collected samples applied to FTA cards in women from a community-based clinic and a referral clinic in Eastern Cape, South Africa. We observed high HR-HPV prevalence, overall agreement of HR-HPV infection, and a similar distribution of HR-HPV genotypes between self-collected and clinician-collected samples. A similar trend has been reported using FTA cards and PCR-based assays performed in women from the general population and referral population [23, 26, 31]. Our findings confirm that the self-collection method and storage on FTA cards is an adequate procedure for HPV DNA testing and support the use of self-collection as an alternative strategy in the regular CC screening routine. Many developed countries have transitioned to HPV DNA testing, and CC screening guidelines with HPV DNA testing has been implemented [17, 18, 32]. However, this is the opposite for low-income countries having low participation rate in CC screening particularly in rural regions. Therefore, introducing new screening strategies for women experiencing screening barriers will improve uptake and could have an impact on the incidence of CC diseases [33].

The prevalence of HR-HPV infection and distribution of HR-HPV genotypes differs between regions and countries [1, 34]. In the current study, HPV16 and HPV35 were the most frequently detected genotypes in both self-collected and clinician-collected samples in the two sites. Other cross-sectional studies conducted in South Africa also reported HPV35 and HPV16 as the most commonly identified genotypes among women with or without cervical abnormalities [35–37]. However, the predominate HR-HPV genotypes in other African studies were HPV18 & HPV59 in Ghana and HPV52 & HPV16 in Tanzania [35, 38]. A recent study done in Brazil among women residing in rural areas showed HPV56 and HPV51 the common types in their population [39]. The two HR-HPV genotypes (HPV16 and 35) are among the top five causing CC in South Africa [3]. None of the HPV vaccines protect against HPV35 (overall prevalence = 18.5%, mostly in multiple infections); an important HR-HPV genotype in women with cervical disease in the Eastern Cape. A high proportion of 72.0% CIN3/CC women were more likely to harbour vaccine HR-HPV types (HPV16/18/31/33/45/52/58). Our results indicate that studies on the distribution of HPV genotypes in CC not present in the current vaccines should be considered when developing strategies and targets for the next generation of HPV vaccines.

A slightly higher prevalence of multiple HR-HPV infections was observed in self-collected as compared to clinician-collected samples in the community-based clinic which could reflect the anatomic area sampled. Vaginal self-collected samples contain vaginal fluid with both cervical and vaginal cells. Women are more likely to be infected with several HPV genotypes in the vagina that are not present in the endocervix [22, 31]. HR-HPV genotypes at low viral load present only in vaginal self-collected samples are not correlated with CC diseases [22, 40]. The few cases of discordance of HPV16 between self-collected and clinician-collected samples among women with CIN3 in our study, were likely to be due to low viral load.

There was high concordance in HR-HPV detection between self-collected and clinician-collected samples in the present study. The use of the Viba-brush in combination with FTA cards, yields good quality DNA in both self-collected and clinician-collected specimen, indicating that the self-collection was performed correctly. Sampling devices for HPV DNA testing have a strong effect on the detection of HPV infection. Self-sampling with a brush-based sampling device in combination with FTA cards was found to be acceptable among women and this together with the clinically validated PCR-based HPV DNA test yielded high agreement in identifying women with high risk of CIN2+ in comparison with clinician-collection sampling [31]. The in-house *hpVIR* assay combined with the FTA cards performed well compared to HC-2, as demonstrated by substantial agreement in HR-HPV positivity. Our findings concur with previous studies reporting on the performance of the two assays, indicating that the *hpVIR* assay and FTA cards can be utilised for primary screening programmes in regions other than Sweden [29, 30].

At both study sites, women showed the same attitude regarding self-collection versus clinican collection samples for HPV DNA testing. The high rate of willingness to perform self-collection was not influenced by sociodemographic factors or any other factor studied as observed in previous studies [41, 42]. This underscores the strong scientific basis for implementing self-collection in CC screening, thereby enabling women from communities and rural settings where CC screening programmes are presently not available to participate. Moreover, the high rate of acceptability for vaginal self-collection could influence the effectiveness of HPV self-collection in routine screening programme [43]. Although self-collection was highly accepted, the majority of women preferred the specimen to be taken by a healthcare worker. In our study, we did not investigate factors influencing women’s choice as to why they prefer clinician-collection of samples. Mao and colleagues (2017) reported that women who prefer a clinician-collection want to have a one-on-one consultation with a clinician, so that they can address other issues they may have [44]. Other factors that women are raising include adequacy of the specimen and lack of confidence to perform self-collection correctly [42, 45]. Therefore, our findings suggest that women should be given an opportunity to choose which method they prefer for specimen collection, when participating in the CC screening programme.

A large population of women in the Eastern Cape Province reside in rural areas and are less likely to participate in CC screening programme because of the lack of nearby facilities with functional CC screening programmes [7]. Implementing self-sampling in this region will therefore have an impact on the screening programme since it does not require infrastructure. This approach allows women who are unwilling to attend Pap smear test performed by clinician to have access to cervical screening and give them privacy to collect the specimen [23]. Implementation of self-collection is also more likely to create awareness and more information about HPV infection than clinician-collection [46, 47]. There are different methods to administer the self-collection kit, including mailing the kit directly to the woman, conducting community-based campaigns and using an opt-in strategy [19]. In developed countries, such as Sweden, mailing self-collection kits to underscreened women has been shown to work effectively [33]. However, in South Africa, it will be challenging to send self-collection kits as the postal system is not working effectively or does not exist in some rural areas. Direct offer of self-collection kit would be a better method to achieve knowledge and confidence among women residing in rural communities as this method has been previously shown to have significantly high rate (>75%) among underscreened women [19]. A second issue would be loss of women to follow-up due to difficulity in travelling to the clinics, something which would have a strong negative impact on the efficiency of CC screening programmes. Therefore, effective communication strategies to follow-up women with HR-HPV positive test needs to be developed prior to implemention of self-collection for HPV testing outside the clinics in South Africa.

The FTA cards provide several advantages for self-collection in this environment. These strategies have the potential to improve CC screening programmes by increasing the screening coverage, reducing the work load for the clinicians, and identify women at high-risk of developing CC [19, 33]. Furthermore, this allows healthcare facilities with limited resources for CC screening to target women at high-risk of cervical cancer. However, self-collection for HPV has been found to increase the rate of women being referred to colposcopy clinics and for treatment. Biomarkers such as DNA methylation has been reported to triage self-collected HR-HPV positive women, but further studies are needed to determine its clinical value [48]. Repeating the HPV test in 4–6 months for women that are HPV positive in their screening test, can be used to identify women with persistent infection and thereby increase the sensitivity and specificity of screening [33]. If it was possible to rescreen women this may be the best approach to triage women for referral to the treatment clinic.

A potential limitation in this study could be the use of in-house *hpVIR* real time PCR assay. The *hpVIR* assay is clinically validated HPV test that gives HPV type information, but it does not distinguish between the alpha HPV types, HPV18/45 and HPV33/52/58 because they are closely related. This limits the ability to provide the prevalence of the individual HPV types that are grouped in the *hpVIR* assay.

## Conclusion

The study shows a high concordance in HR-HPV genotype prevalence between self-collected and clinician-collected specimens. The frequency distribution of HR-HPV genotypes will assist in identifying and monitoring genotypes that are not covered by the vaccines currently in use. The self-collection procedure for HPV testing was regarded as highly acceptable by the women. Self-collection can have a positive impact on the cervical screening programme in South Africa by increasing the population coverage in rural areas, and enable women who are unable to attend clinics to participate in cervical cancer screening.

## Supporting information

S1 Data (CSV)

S2 Data (CSV)

## Abbreviations

CC: cervical cancer
CI: confidence intervals, ref: reference
CIN: cervical intraepithelial neoplasia
HR-HPV: high-risk human papillomavirus
NILM: negative for intraepithelial lesions or malignancy
VIA: Visual Inspection Acetic acid
